# Primary Peritoneal Drainage Versus Laparotomy for Perforated Necrotizing Enterocolitis in Very-Low-Birth-Weight Infants: A Retrospective Cohort Study at an Academic Center in Saudi Arabia

**DOI:** 10.7759/cureus.33895

**Published:** 2023-01-17

**Authors:** Heidi Al-Wassia, Maha Bamehrez, Hisham A Basamh, Mohanned S Aljohany, Nasir M Bustangi

**Affiliations:** 1 Pediatrics, King Abdulaziz University Hospital, Jeddah, SAU; 2 Neonatology, King Abdulaziz University Hospital, Jeddah, SAU; 3 Pediatric Surgery, King Abdulaziz University Hospital, Jeddah, SAU; 4 Pediatric Surgery, Armed Forces Hospital, Southwest Region, Khamis Mushait, SAU

**Keywords:** primary peritoneal drainage, intestinal perforation, perforation, vlbw, exploration, laparotomy, peritoneal drainage, necrotizing enterocolitis, nec

## Abstract

Background and objective

Necrotizing enterocolitis (NEC) is a detrimental complication of the gastrointestinal tract among preterm infants with very low birth weight (VLBW) and is associated with high morbidity and mortality. About one-third of these cases require surgical intervention due to intestinal perforation. The preferred method for the surgical management of perforated NEC is still a matter of controversy. In light of this, we aimed to compare the outcomes of treating perforated NEC in VLBW infants with primary peritoneal drainage (PPD) versus laparotomy.

Method

We conducted a retrospective chart review of VLBW infants with perforated NEC treated at King Abdulaziz University Hospital between January 1, 2015, and March 31, 2020.

Results

Twenty-seven infants with perforated NEC were identified; 12 were managed initially with PPD, and 15 underwent laparotomy. There was no difference between groups in terms of postoperative outcomes, length of hospital stay, or mortality before discharge. Among infants managed with PPD, 50% (5/10) underwent second drainage and survived, while 33% (4/12) underwent laparotomy.

Conclusion

We identified no difference in postoperative outcomes and mortality between managing perforated NEC in VLBW infants with either PPD or laparotomy. However, randomized clinical trials with larger sample sizes and defined outcome measures are needed for reaching definitive conclusions.

## Introduction

Necrotizing enterocolitis (NEC) is the most common gastrointestinal/surgical emergency among preterm infants, with an incidence of 7-8% in very-low-birth-weight (VLBW) infants (less than 1500 grams) and an associated mortality reaching as high as 30-40% [1]. In a multicenter retrospective analysis of 2,948 extremely low-birth-weight (ELBW) infants (<1000 grams), surgically managed NEC was associated with significant adverse neurodevelopmental outcomes (27/46, 58.7%) at 18-22 months [2].

The surgical procedures performed in cases of NEC are either laparotomy and bowel resection or bedside primary peritoneal drainage (PPD). Surgical intervention in patients with NEC mainly manages enteric leakage and/or resects the necrotic intestine [3]. Although laparotomy is the traditional surgical management for perforated NEC, PPD is the preferred initial procedure in ELBW infants [4]. However, the appropriate type of surgical management for NEC in VLBW infants is still controversial, as there is limited data to support the superiority of one procedure over the other. In a Cochrane systematic review and meta-analysis involving preterm infants (<34 weeks' gestation) with perforated NEC, there was no significant difference in mortality, need for total parenteral nutrition (TPN), or length of hospital stay between infants managed with PPD and those who underwent laparotomy [5]. The surgical management of perforated NEC has not been previously studied at our center. Hence, we conducted this study with a view to comparing the short-term outcomes between VLBW infants managed with PPD and those managed with laparotomy for perforated NEC.

## Materials and methods

Study design

This was a retrospective cohort study conducted at King Abdulaziz University Hospital (KAUH), Jeddah. The Institutional Research Ethics Board at KAUH approved the study and waived informed consent for all included patients (reference no: 319-21).

Population and setting

KAUH is a tertiary academic center with a 34 neonatal intensive care unit (NICU)-bed capacity. Four pediatric surgeons and rotating pediatric surgery fellows cover the pediatric surgery service. Our sample included all preterm infants (<1500 grams) with stage III NEC according to modified Bell's staging criteria who underwent surgical intervention between January 1, 2015, and March 31, 2020. We excluded infants with spontaneous intestinal perforation, abdominal wall defects, cyanotic congenital heart disease, and major congenital anomalies. Collected maternal characteristics included whether the mother was booked (two or more antenatal visits), nationality (Saudi or not), hypertension (gestational or chronic), diabetes (pregnancy-induced or other types), and antenatal steroid use (at least one dose before delivery). Infant characteristics that were gathered included gestational age in weeks (based on the first day of the last menstrual period or early ultrasonography), the need for resuscitation (chest compression and medications), and sepsis (defined as positive blood culture).

Study outcomes

The primary outcome was mortality before discharge. We also collected information about the day of postoperative mortality. Secondary outcomes included the length of hospital stay till discharge or death, total postoperative days on TPN, the day of starting enteral feeding, the time needed to reach full feeding, total duration of invasive mechanical ventilation, development of strictures, and the need for laparotomy if PPD was the initial management modality.

Statistical analysis

All statistical analyses were done using IBM SPSS Statistics for Windows, version 21 (IBM Corp., Armonk, NY). Continuous variables are presented as means and standard deviations (SD) or median and interquartile range (IQR) and compared using the student's t-test or Wilcoxon rank test. Categorical variables are depicted using frequencies and percentages and compared utilizing the chi-square test. P-values <0.05 were considered statistically significant.

## Results

A total of 27 cases were included in this study, of which 15 (56%) were managed with laparotomy, and 12 (44%) had PPD. There was no significant difference in the age at NEC diagnosis between groups. Also, there were no significant differences in maternal and infant characteristics between the groups (Table 1).

**Table 1:** Baseline characteristics of infants treated with peritoneal drainage vs. laparotomy Results are presented as mean (SD), median (IQR), or frequency (%) IQR: interquartile range; NEC: necrotizing enterocolitis; SD: standard deviation

Variable	Laparotomy (n=15)	Drainage (n=12)	P-value
Maternal characteristics			
Booked	6 (40%)	7 (58%%)	0.29
Saudi National	7 (47%)	8 (67)	0.26
Hypertension	1 (7%)	0 (0%)	0.56
Diabetes	1 (7%)	2 (17%)	0.41
Antenatal steroid use	8 (53%)	9 (75%)	0.23
Neonatal characteristics			
Male	8 (53%)	6 (50%)	0.59
Female	7 (47%)	6 (50%)	
Gestational age, weeks	28 (2.7%)	27 (1.5%)	0.50
Birth weight, grams	941 (232)	932 (289)	0.91
Need for resuscitation at birth	4 (29%)	3 (25%)	0.59
Sepsis	13 (86.7%)	7 (63.6%)	0.35
Age at NEC diagnosis, days	12 (8.5, 14.5)	13 (8.5, 16.5)	0.69

Two infants died less than 24 hours after undergoing PPD. There was no significant difference in the median TPN duration and days to reach full feeding between the groups (Table 2).

**Table 2:** Postoperative outcomes of laparotomy vs. peritoneal drainage Results are presented as mean (SD), median (IQR), or frequency (%) IQR: interquartile range; SD: standard deviation; TPN: total parenteral nutrition

Variable	Laparotomy (n=15)		Drainage (n=12)		P-value
Days to start feeding	11 (10, 14)	(n=9)	12 (11, 13)	(n=8)	1.00
Number of days on TPN	15 (10, 23)	(n=11)	10 (2.5, 21)	(n=10)	0.67
Days to reach full feeding	21 (19, 27)	(n=9)	22 (19, 25)	(n=7)	1.00
Number of days on mechanical ventilation	12 (6.5, 16)		12.5 (7, 30)	(n=10)	1.00
Number of infants requiring a second drainage	-		5 (50%)	(n=10)	
Number of infants requiring laparotomy after drainage			4 (33%)		
Day of laparotomy after drainage	-		5.5 (2.5, 9)		
Number of infants developing strictures	0 (0)		0 (0)		-
Length of hospital stay, days	75 (12, 313)		38 (8, 132)		0.21
Mortality before discharge	7 (47%)		5 (42%)		1.00
Day of postoperative mortality	76 (54, 121)		29 (14, 38)		0.32

Five infants (50%) in the PPD group needed second drainage, while four (33%) underwent laparotomy. None of the included infants developed strictures. There was no difference in in-hospital mortality or median hospital stay duration between the groups.

## Discussion

We retrospectively compared the outcomes of PPD and laparotomy for the treatment of perforated NEC in 27 VLBW infants. We identified no significant difference between the two groups in short-term outcomes and mortality. Similar to most other published studies, the infants in the PPD group were lower in gestational age and smaller in weight. However, this difference was not statistically significant, most likely due to the small sample size of our study [6,7].

In 1977, Ein et al. reported on managing intestinal perforation in five patients with PPD [8]. Three of the five patients survived, and one required a reoperation. Three years later, Janik and Ein reported complete recovery after surgical NEC without needing a follow-up laparotomy in 40% of patients managed initially with PPD [9]. Since then, PPD has been widely acknowledged as the treatment of choice for preterm infants with perforated NEC [10-12].

A shorter period to achieve full enteral feeding and reduced reliance on parenteral nutrition are favored outcomes because prolonged TPN duration is associated with an increased risk of catheter-related sepsis and other morbidities [13]. The presence of the necrotic intestine and accompanying inflammation may delay the initiation and advancement of enteral feeds and explain why infants who are managed with PPD take longer to attain enteral feeding [5]. Moreover, with PPD, the necrotic intestines release inflammatory mediators, which are linked to poor neurodevelopmental outcomes [14]. In our study, there was no statistically significant difference between groups in terms of total TPN duration or days to reach full feeding. In a multicenter randomized clinical trial involving 117 VLBW infants with perforated NEC, TPN duration and days to reach full enteral feeding were not found to be different between infants managed with PPD and those who underwent laparotomy and bowel resection [15].

In our study, 33% of infants managed with PPD required a laparotomy within a median of 5.5 (2.5, 9) days. Infants who undergo emergency laparotomies are more likely to experience intraoperative and postoperative complications [5]. Supporters of PPD may also claim that the ability to avoid inhalational anesthesia and fluid shifts that put VLBW infants at greater risk for cardiopulmonary compromise is a significant advantage.

In a multicenter international RCT involving preterm infants ≥1000 grams allocated to either laparotomy or PPD group, deferred laparotomy did not improve the six-month survival compared with primary laparotomy [16]. In the study by Moss et al., 5/55 (9%) of patients had to undergo a delayed laparotomy due to a lack of clinical improvement, and 16/55 (29%) patients underwent the same due to intestinal complications such as stricture, bowel obstruction, or intolerance to feeding [15]. Peritoneal drainage is mostly used in smaller and unstable infants due to the belief that they may not tolerate laparotomy. Furthermore, PPD has been used as a temporizing procedure followed by laparotomy. In a retrospective chart review of 42 ELBW infants with pneumoperitoneum, infants managed with PPD had significantly lower birth weights and gestational age [7]. In the same study, 38% of infants with PPD avoided a laparotomy. They had comparable outcomes with infants managed with primary laparotomy in terms of mortality within 90 days, time to full enteral feeding, and duration of hospital stay [7]. In a retrospective cohort study of 50 infants with pneumoperitoneum with birthweight <1800 and gestational age <33 weeks, 12 (32%) of the 38 infants who underwent initial PPD required a secondary laparotomy [17]. There was no statistically significant difference in mortality between groups; however, the hazard ratio for death in the PPD group was partly confounded by birthweight [17].

There was no difference between groups in terms of total TPN duration and time to reach the full feed in the present study. In a large retrospective cohort study involving 528 infants with <32 weeks’ gestation and <1000 grams with surgical NEC, primary laparotomy was the initial procedure in 68% of infants; infants receiving LAP were older and heavier and had fewer incidence of severe intraventricular hemorrhage [18]. In the same study, survivors who had undergone laparotomy as the initial surgical approach were more likely to develop short bowel syndrome. The association remained significant even after adjusting for identified confounders. However, like our study, there was no significant difference between the two intervention groups regarding mortality as a single or composite outcome with short bowel syndrome [18]. Likewise, in the two RCTs by Moss et al. and Rees et al., there was no significant difference in survival between the drain and primary laparotomy groups [15,16]. In a multicenter prospective cohort study of 156 ELBW infants with severe NEC or spontaneous intestinal perforation (SIP), 80 were treated with PPD, and 76 had initial laparotomy [6]. Initial laparotomy had a non-significant lower mortality risk alone or as a component of composite outcomes with short bowel syndrome or neurodevelopmental impairment at 18-22 months. The risk increased after adjustment for potential confounders as the infants who received initial drainage were relatively smaller and sicker [6]. In a recent and large RCT conducted at 20 US centers, which included 310 infants ≤1000 grams with the initial diagnosis of NEC or intestinal perforation, there was no difference in the rates of death or neurodevelopmental impairment at the corrected age of 18-22 months between the initial PPD and laparotomy groups. Furthermore, initial laparotomy was more likely than initial drainage to reduce death or neurodevelopmental impairment, implying an effect modification by preoperative diagnosis [19].

This study has a few limitations that need to be addressed. The retrospective design and the small sample size were a limitation in terms of reaching a valid conclusion regarding the comparison between the two interventions. Moreover, the study was not powered enough either to detect differences of smaller magnitudes or to carry out a regression analysis to correct for confounders. Also, we only collected information about sepsis and the need for resuscitation to indicate how sick the infants were; nevertheless, we did not have further details on the severity of NEC to account for confounding by indication. However, we included all VLBW infants born in KAUH and diagnosed with perforated NEC in the specified study period, which minimizes selection bias.

## Conclusions

Our study identified no significant differences in postoperative outcomes or mortality between VLBW infants with perforated NEC managed with PPD and those treated with laparotomy. Moreover, there were no significant statistical differences between the two groups regarding initiation of enteral feeds, TPN duration, and days to reach full enteral feeding. Further randomized clinical trials with a larger study population and defined outcomes are needed to synthesize research data and contribute to knowledge translation.
